# Supplementary material for: HIVprotI: an integrated web based platform for prediction and design of HIV proteins inhibitors
Source: J Cheminform. 2018 Mar 9;10:12. doi: 10.1186/s13321-018-0266-y (PMC5845081; doi:10.1186/s13321-018-0266-y)
Supplement: Supplementary file 2 — Additional file 2. Source code of HIVProtI web server. [file 13321_2018_266_MOESM2_ESM.zip › HIVprotI_Source-code/hivprotI/weka-3-6-6/doc/weka/core/Matrix.html]

Matrix


---


|  |  |  |
| --- | --- | --- |
| **Overview** \| **Package** \| **Class** \| **Tree** \| **Deprecated** \| **Index** \| **Help** \| **Weka's home** | |  |
| **PREV CLASS**   **NEXT CLASS** | **FRAMES**    **NO FRAMES**     **All Classes** |  |
| SUMMARY: NESTED \| FIELD \| CONSTR \| METHOD | DETAIL: FIELD \| CONSTR \| METHOD |  |


---


## weka.core Class Matrix

```
java.lang.Object
  weka.core.Matrix
```

**All Implemented Interfaces:**: java.io.Serializable, java.lang.Cloneable, RevisionHandler

**Direct Known Subclasses:**: ConfusionMatrix

---

**Deprecated.** *Use `weka.core.matrix.Matrix` instead - only for
backwards compatibility.*

``` public class Matrix extends java.lang.Object implements java.lang.Cloneable, java.io.Serializable, RevisionHandler ```

Class for performing operations on a matrix of floating-point values.

Deprecated: Uses internally the code of the sub-package
`weka.core.matrix` - only for backwards compatibility.

**Version:**
:   $Revision: 1.25 $

**Author:**
:   Gabi Schmidberger (gabi@cs.waikato.ac.nz), Yong Wang (yongwang@cs.waikato.ac.nz), Eibe Frank (eibe@cs.waikato.ac.nz), Len Trigg (eibe@cs.waikato.ac.nz), Fracpete (fracpete at waikato dot ac dot nz)

**See Also:**: Serialized Form

---

**Constructor Summary**

`Matrix(double[][] array)`
  
          **Deprecated.** Constructs a matrix using a given array.

`Matrix(int nr,
int nc)`
  
          **Deprecated.** Constructs a matrix and initializes it with default values.

`Matrix(java.io.Reader r)`
  
          **Deprecated.** Reads a matrix from a reader.


| **Method Summary** | |
| --- | --- |
| `Matrix` | `add(Matrix other)`             **Deprecated.** Returns the sum of this matrix with another. |
| `void` | `addElement(int rowIndex, int columnIndex, double value)`             **Deprecated.** Add a value to an element. |
| `java.lang.Object` | `clone()`             **Deprecated.** Creates and returns a clone of this object. |
| `void` | `eigenvalueDecomposition(double[][] V, double[] d)`             **Deprecated.** Performs Eigenvalue Decomposition using Householder QR Factorization Matrix must be symmetrical. |
| `double[]` | `getColumn(int index)`             **Deprecated.** Gets a column of the matrix and returns it as a double array. |
| `double` | `getElement(int rowIndex, int columnIndex)`             **Deprecated.** Returns the value of a cell in the matrix. |
| `Matrix` | `getL()`             **Deprecated.** Returns the L part of the matrix. |
| `java.lang.String` | `getRevision()`             **Deprecated.** Returns the revision string. |
| `double[]` | `getRow(int index)`             **Deprecated.** Gets a row of the matrix and returns it as double array. |
| `Matrix` | `getU()`             **Deprecated.** Returns the U part of the matrix. |
| `boolean` | `isSymmetric()`             **Deprecated.** Returns true if the matrix is symmetric. |
| `int[]` | `LUDecomposition()`             **Deprecated.** Performs a LUDecomposition on the matrix. |
| `static void` | `main(java.lang.String[] ops)`             **Deprecated.** Main method for testing this class. |
| `Matrix` | `multiply(Matrix b)`             **Deprecated.** Returns the multiplication of two matrices |
| `int` | `numColumns()`             **Deprecated.** Returns the number of columns in the matrix. |
| `int` | `numRows()`             **Deprecated.** Returns the number of rows in the matrix. |
| `static Matrix` | `parseMatlab(java.lang.String matlab)`             **Deprecated.** creates a matrix from the given Matlab string. |
| `double[]` | `regression(Matrix y, double ridge)`             **Deprecated.** Performs a (ridged) linear regression. |
| `double[]` | `regression(Matrix y, double[] w, double ridge)`             **Deprecated.** Performs a weighted (ridged) linear regression. |
| `void` | `setColumn(int index, double[] newColumn)`             **Deprecated.** Sets a column of the matrix to the given column. |
| `void` | `setElement(int rowIndex, int columnIndex, double value)`             **Deprecated.** Sets an element of the matrix to the given value. |
| `void` | `setRow(int index, double[] newRow)`             **Deprecated.** Sets a row of the matrix to the given row. |
| `void` | `solve(double[] bb)`             **Deprecated.** Solve A\*X = B using backward substitution. |
| `java.lang.String` | `toMatlab()`             **Deprecated.** converts the Matrix into a single line Matlab string: matrix is enclosed by parentheses, rows are separated by semicolon and single cells by blanks, e.g., [1 2; 3 4]. |
| `java.lang.String` | `toString()`             **Deprecated.** Converts a matrix to a string |
| `Matrix` | `transpose()`             **Deprecated.** Returns the transpose of a matrix. |
| `void` | `write(java.io.Writer w)`             **Deprecated.** Writes out a matrix. |

**Methods inherited from class java.lang.Object**

`equals, getClass, hashCode, notify, notifyAll, wait, wait, wait`

**Constructor Detail**

### Matrix

```
public Matrix(int nr,
              int nc)
```

:   **Deprecated.**:   Constructs a matrix and initializes it with default values.

        **Parameters:**: `nr` - the number of rows: `nc` - the number of columns

---


### Matrix

```
public Matrix(double[][] array)
       throws java.lang.Exception
```

:   **Deprecated.**:   Constructs a matrix using a given array.

        **Parameters:**: `array` - the values of the matrix **Throws:**: `java.lang.Exception`

---


### Matrix

```
public Matrix(java.io.Reader r)
       throws java.lang.Exception
```

:   **Deprecated.**:   Reads a matrix from a reader. The first line in the file should
        contain the number of rows and columns. Subsequent lines
        contain elements of the matrix.

        **Parameters:**: `r` - the reader containing the matrix **Throws:**: `java.lang.Exception` - if an error occurs


**Method Detail**

### clone

```
public java.lang.Object clone()
```

:   **Deprecated.**:   Creates and returns a clone of this object.

        :   **Overrides:**: `clone` in class `java.lang.Object`
        :   **Returns:**: a clone of this instance. **Throws:**: `java.lang.Exception` - if an error occurs

---


### write

```
public void write(java.io.Writer w)
           throws java.lang.Exception
```

:   **Deprecated.**:   Writes out a matrix.

        :   **Parameters:**: `w` - the output Writer **Throws:**: `java.lang.Exception` - if an error occurs

---


### getElement

```
public final double getElement(int rowIndex,
                               int columnIndex)
```

:   **Deprecated.**:   Returns the value of a cell in the matrix.

        :   **Parameters:**: `rowIndex` - the row's index: `columnIndex` - the column's index **Returns:**: the value of the cell of the matrix

---


### addElement

```
public final void addElement(int rowIndex,
                             int columnIndex,
                             double value)
```

:   **Deprecated.**:   Add a value to an element.

        :   **Parameters:**: `rowIndex` - the row's index.: `columnIndex` - the column's index.: `value` - the value to add.

---


### numRows

```
public final int numRows()
```

:   **Deprecated.**:   Returns the number of rows in the matrix.

        :   **Returns:**: the number of rows

---


### numColumns

```
public final int numColumns()
```

:   **Deprecated.**:   Returns the number of columns in the matrix.

        :   **Returns:**: the number of columns

---


### setElement

```
public final void setElement(int rowIndex,
                             int columnIndex,
                             double value)
```

:   **Deprecated.**:   Sets an element of the matrix to the given value.

        :   **Parameters:**: `rowIndex` - the row's index: `columnIndex` - the column's index: `value` - the value

---


### setRow

```
public final void setRow(int index,
                         double[] newRow)
```

:   **Deprecated.**:   Sets a row of the matrix to the given row. Performs a deep copy.

        :   **Parameters:**: `index` - the row's index: `newRow` - an array of doubles

---


### getRow

```
public double[] getRow(int index)
```

:   **Deprecated.**:   Gets a row of the matrix and returns it as double array.

        :   **Parameters:**: `index` - the row's index **Returns:**: an array of doubles

---


### getColumn

```
public double[] getColumn(int index)
```

:   **Deprecated.**:   Gets a column of the matrix and returns it as a double array.

        :   **Parameters:**: `index` - the column's index **Returns:**: an array of doubles

---


### setColumn

```
public final void setColumn(int index,
                            double[] newColumn)
```

:   **Deprecated.**:   Sets a column of the matrix to the given column. Performs a deep copy.

        :   **Parameters:**: `index` - the column's index: `newColumn` - an array of doubles

---


### toString

```
public java.lang.String toString()
```

:   **Deprecated.**:   Converts a matrix to a string

        :   **Overrides:**: `toString` in class `java.lang.Object`
        :   **Returns:**: the converted string

---


### add

```
public final Matrix add(Matrix other)
```

:   **Deprecated.**:   Returns the sum of this matrix with another.

        :   **Returns:**: a matrix containing the sum.

---


### transpose

```
public final Matrix transpose()
```

:   **Deprecated.**:   Returns the transpose of a matrix.

        :   **Returns:**: the transposition of this instance.

---


### isSymmetric

```
public boolean isSymmetric()
```

:   **Deprecated.**:   Returns true if the matrix is symmetric.

        :   **Returns:**: boolean true if matrix is symmetric.

---


### multiply

```
public final Matrix multiply(Matrix b)
```

:   **Deprecated.**:   Returns the multiplication of two matrices

        :   **Parameters:**: `b` - the multiplication matrix **Returns:**: the product matrix

---


### regression

```
public final double[] regression(Matrix y,
                                 double ridge)
```

:   **Deprecated.**:   Performs a (ridged) linear regression.

        :   **Parameters:**: `y` - the dependent variable vector: `ridge` - the ridge parameter **Returns:**: the coefficients **Throws:**: `java.lang.IllegalArgumentException` - if not successful

---


### regression

```
public final double[] regression(Matrix y,
                                 double[] w,
                                 double ridge)
```

:   **Deprecated.**:   Performs a weighted (ridged) linear regression.

        :   **Parameters:**: `y` - the dependent variable vector: `w` - the array of data point weights: `ridge` - the ridge parameter **Returns:**: the coefficients **Throws:**: `java.lang.IllegalArgumentException` - if the wrong number of weights were provided.

---


### getL

```
public Matrix getL()
            throws java.lang.Exception
```

:   **Deprecated.**:   Returns the L part of the matrix.
        This does only make sense after LU decomposition.

        :   **Returns:**: matrix with the L part of the matrix; **Throws:**: `java.lang.Exception` **See Also:**: `LUDecomposition()`

---


### getU

```
public Matrix getU()
            throws java.lang.Exception
```

:   **Deprecated.**:   Returns the U part of the matrix.
        This does only make sense after LU decomposition.

        :   **Returns:**: matrix with the U part of a matrix; **Throws:**: `java.lang.Exception` **See Also:**: `LUDecomposition()`

---


### LUDecomposition

```
public int[] LUDecomposition()
                      throws java.lang.Exception
```

:   **Deprecated.**:   Performs a LUDecomposition on the matrix.
        It changes the matrix into its LU decomposition.

        :   **Returns:**: the indices of the row permutation **Throws:**: `java.lang.Exception`

---


### solve

```
public void solve(double[] bb)
           throws java.lang.Exception
```

:   **Deprecated.**:   Solve A\*X = B using backward substitution.
        A is current object (this). Note that this matrix will be changed!
        B parameter bb.
        X returned in parameter bb.

        :   **Parameters:**: `bb` - first vector B in above equation then X in same equation. **Throws:**: `java.lang.Exception`

---


### eigenvalueDecomposition

```
public void eigenvalueDecomposition(double[][] V,
                                    double[] d)
                             throws java.lang.Exception
```

:   **Deprecated.**:   Performs Eigenvalue Decomposition using Householder QR Factorization
        Matrix must be symmetrical.
        Eigenvectors are return in parameter V, as columns of the 2D array.
        (Real parts of) Eigenvalues are returned in parameter d.

        :   **Parameters:**: `V` - double array in which the eigenvectors are returned: `d` - array in which the eigenvalues are returned **Throws:**: `java.lang.Exception` - if matrix is not symmetric

---


### toMatlab

```
public java.lang.String toMatlab()
```

:   **Deprecated.**:   converts the Matrix into a single line Matlab string: matrix is enclosed
        by parentheses, rows are separated by semicolon and single cells by
        blanks, e.g., [1 2; 3 4].

        :   **Returns:**: the matrix in Matlab single line format

---


### parseMatlab

```
public static Matrix parseMatlab(java.lang.String matlab)
                          throws java.lang.Exception
```

:   **Deprecated.**:   creates a matrix from the given Matlab string.

        :   **Parameters:**: `matlab` - the matrix in matlab format **Returns:**: the matrix represented by the given string **Throws:**: `java.lang.Exception` **See Also:**: `toMatlab()`

---


### getRevision

```
public java.lang.String getRevision()
```

:   **Deprecated.**:   Returns the revision string.

        :   **Specified by:**: `getRevision` in interface `RevisionHandler`
        :   **Returns:**: the revision

---


### main

```
public static void main(java.lang.String[] ops)
```

:   **Deprecated.**:   Main method for testing this class.


---


|  |  |  |
| --- | --- | --- |
| **Overview** \| **Package** \| **Class** \| **Tree** \| **Deprecated** \| **Index** \| **Help** \| **Weka's home** | |  |
| **PREV CLASS**   **NEXT CLASS** | **FRAMES**    **NO FRAMES**     **All Classes** |  |
| SUMMARY: NESTED \| FIELD \| CONSTR \| METHOD | DETAIL: FIELD \| CONSTR \| METHOD |  |


---
